# Long-term outcomes of kidney transplantation in two recipients from a single membranous nephropathy donor: Case reports and literature review

**DOI:** 10.1016/j.ijscr.2025.111520

**Published:** 2025-06-14

**Authors:** Shan-Qun Shen, Wen-Han Peng

**Affiliations:** a Kidney Disease Center, the First Affiliated Hospital, College of Medicine, Zhejiang University, Hangzhou 310003, Zhejiang, China; b Key Laboratory of Kidney Disease Prevention and Control Technology, Hangzhou 310003, Zhejiang, China; c National Key Clinical Department of Kidney Diseases, Hangzhou 310003, Zhejiang, China; d Institute of Nephrology, Zhejiang University, Hangzhou 310003, Zhejiang, China; e The Third Grade Laboratory under the National State, Administration of Traditional Chinese Medicine, Hangzhou 310003, Zhejiang, China

**Keywords:** Kidney transplantation, Donor shortage, Membranous nephropathy donor, Long-term prognosis, Case report

## Abstract

**Introduction and importance:**

Kidney transplantation encounters a significant challenge due to the persistent shortage of donor organs. Membranous nephropathy (MN) is a rare entity among donor-derived glomerular diseases, and its impact on allograft function and long-term survival remains incompletely understood.

**Case presentation:**

We report two kidney transplants from a donor with early-stage MN. The left kidney recipient developed significant proteinuria and elevated creatinine, with a biopsy confirming acute T cell-mediated rejection and donor-derived MN. Following short-term peritoneal dialysis and intensified immunosuppression, serum creatinine levels progressively declined, leading to a restoration of renal function. The right kidney recipient experienced an uncomplicated postoperative course and maintained stable renal function. 8-month protocol biopsies in both recipients demonstrated partial pathological remission. At the 8-year follow-up post-transplantation, both recipients maintained excellent renal function.

**Clinical discussion:**

Despite the potential risks of proteinuria and dysfunction, and challenges in donor diagnosis associated with MN donor kidneys, our two cases demonstrate excellent long-term graft function (8-year follow-up), even in one recipient who experienced acute rejection that responded to intensified immunosuppression. These observations suggest the potential for carefully selected MN donor kidneys to expand the donor pool, though larger-scale studies are essential given the limitations of a case report.

**Conclusion:**

In these two cases, kidney transplantations from early-stage MN donors exhibited excellent long-term graft function and partial pathological remission. Such results suggest a potential viable strategy to expand the donor pool, contingent upon rigorous clinical oversight and tailored management strategies.

## Highlights

• Kidneys from donors with membranous nephropathy are potentially suitable for transplantations.
• Short-term low-level proteinuria post-transplantation is considered harmless.
• Strategic immunosuppression and protocol biopsies are critical for successful kidney transplantation.

## 1 Introduction

Kidney transplantation faces a significant challenge due to the ongoing shortage of donor organs. The utilization of expanded criteria donor kidneys has broadened the donor pool, thereby increasing transplantation opportunities for a larger number of patients suffering from uremia. Documented short-term outcomes indicate some success in kidney transplantations from donors with preexisting membranous nephropathy (MN). However, long-term graft function remains a crucial determinant of transplant success, and evidence supporting this is currently limited. We present two cases of recipients who received kidneys from the same deceased donor diagnosed with early-stage MN and provide an analysis of previously reported cases. This case report has been reported in line with the 2025 SCARE checklist [1].

## 2 Case presentation

The 50-year-old male donor died from injuries sustained in a car accident. His last recorded serum creatinine level was 46 μmol/L, and urinalysis revealed proteinuria (2+). A donor kidney biopsy conducted at the time of explantation demonstrated immunofluorescence staining for IgG deposits along the glomerular capillary walls, with a strong positivity (3+) for M-type phospholipase A2 receptor (PLA2R). Electron microscopy revealed mild effacement of podocyte foot processes, with no observed thickening of the basement membrane. Further evaluation indicated a circulating PLA2R antibody level of 120 U/mL.

**Fig. 1:**
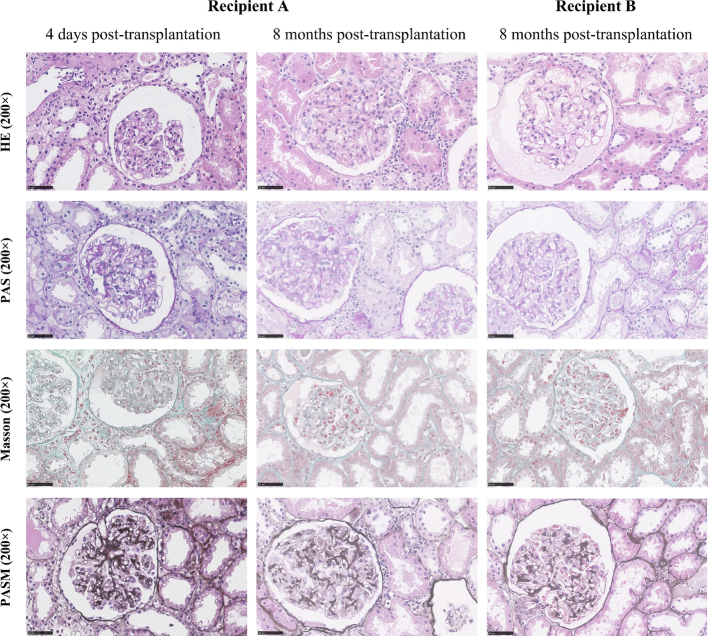
Renal biopsy findings of the recipients.

Recipient A, a 52-year-old male who received the left kidney, had been on peritoneal dialysis for over two years due to chronic renal failure of unknown etiology. Recipient B, a 34-year-old female who received the right kidney, had been on hemodialysis for more than three years. A prior renal biopsy revealed severe chronic tubulointerstitial nephritis, mild mesangial cell proliferation, and global glomerulosclerosis. Pre-transplantation, both recipients were negative for panel reactive antibodies and donor-specific antibodies. On October 10, 2016, both patients underwent kidney transplantation, with warm and cold ischemia times of 5 min and 6 h, respectively. Induction therapy included basiliximab, while maintenance immunosuppression consisted of tacrolimus, mycophenolic acid, and prednisolone. Beginning on postoperative day (POD) 2, Recipient A developed proteinuria exceeding 3 g/day, alongside elevated serum creatinine levels. Sonographic examination of the renal allograft and its vessels revealed increased cortical echogenicity, reduced superficial cortex perfusion, and an elevated intrarenal resistive index. These findings prompted an allograft biopsy, which confirmed acute T cell-mediated rejection (TCMR, Type IIA) characterized by mild to moderate intimal arteritis and minimal interstitial inflammation without tubulitis, as well as donor-derived membranous nephropathy (Fig. 1). Immunofluorescence demonstrated granular IgG deposits along the capillary loops, where immunohistochemistry for PLA2R was strongly positive (3+). Electron microscopy revealed extensive effacement of podocyte foot processes and electron-dense deposits along the basement membrane. Peritoneal dialysis was reinstated. Immunosuppressive therapy was intensified with anti-thymocyte globulin (ATG) at 50 mg daily for four days, followed by a reduction to 25 mg on day 5. This was administered in conjunction with intravenous immunoglobulin (IVIG) at 20 g daily for five days. Following this regimen, serum creatinine levels decreased to 205 μmol/L, corresponding to a glomerular filtration rate of 30.94 mL/min by POD 10. At discharge on POD 14, Recipient A's serum creatinine had further declined to 136 μmol/L, with 24-hour proteinuria of 1.12 g/day. Recipient B experienced an uncomplicated post-transplantation course, demonstrating favorable renal recovery without delayed graft function, acute rejection, or infections. She was discharged on POD 10 with a serum creatinine of 122 μmol/L and 24-hour proteinuria of 0.25 g/day. In March 2022, her immunosuppressive regimen was switched from mycophenolate to sirolimus after resection of thyroid cancer.

**Fig. 2:**
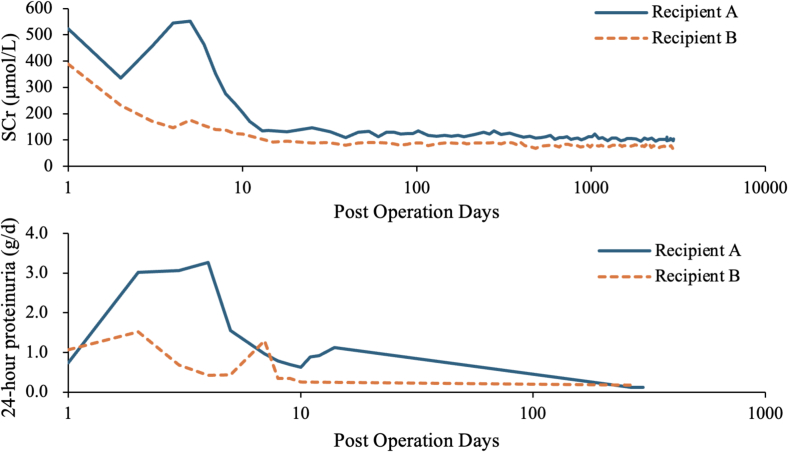
Postoperative trends of the levels of serum creatinine, 24-hour proteinuria, and proteinuria after kidney transplantation.

**Fig. 3:**
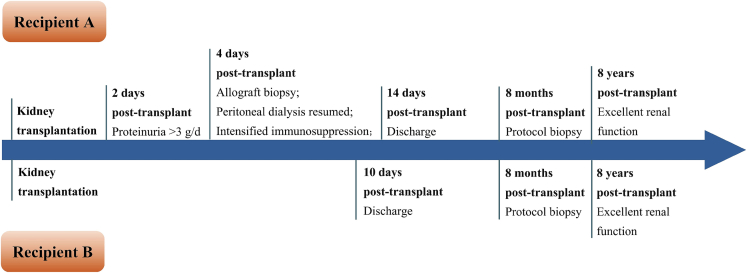
Temporal overview of the postoperative course for both recipients.

The first column depicts mild to moderate intimal arteritis and minimal interstitial inflammation without tubulitis. The basement membrane displays vacuolar degeneration, and no fuchsinophilic deposits are observed; the second and third column illustrates intramembranous vacuolization, tubulitis, minimal interstitial inflammation, and mild proliferation of mesangial cells and matrix. The basement membrane demonstrates vacuolar degeneration, with no fuchsinophilic deposits observed. HE, hematoxylin and eosin staining; PAS, periodic acid-Schiff staining; PASM, periodic scid‑silver methena staining;

At 8 months post-transplantation, protocol biopsies performed on both recipients revealed borderline rejection and membranous nephropathy (MN, Stage I) with accompanying global glomerulosclerosis (Fig. 1). The biopsy of Recipient A included 39 glomeruli, of which one was globally sclerosed (2.56 %). Light microscopy revealed intramembranous vacuolization, tubulitis, trivial interstitial inflammation, and mild proliferation of mesangial cells and matrix. Immunofluorescence staining demonstrated granular global deposits of IgG along the glomerular capillary walls, with PLA2R immunostaining exhibiting moderate positivity (2+). Electron microscopy showed partial effacement of podocyte foot processes and intramembranous electron-dense deposits. The biopsy of Recipient B yielded 37 glomeruli, three of which were globally sclerosed (8.11 %). Light and electron microscopy findings in Recipient B were consistent with those observed in Recipient A. At 8 months post-transplantation, 24-hour urine protein levels were 0.12 g/day for Recipient A and 0.18 g/day for Recipient B. Both recipients tested negative for circulating PLA2R antibodies. Proteinuria remained undetectable in subsequent years (Fig. 2). At 8 years post-transplantation, both recipients maintained excellent renal function, as evidenced by stable serum creatinine levels of 95–110 μmol/L (recipient A) and 65–85 μmol/L (recipient B) (Fig. 3). Further allograft biopsies were not performed due to the absence of clinical indications in either recipient.

This timeline illustrates key clinical events and interventions throughout the 8-year follow-up period. Allograft biopsies were performed for recipient A at 4 days post-transplant, and for both recipient A and recipient B at 8 months post-transplant.

## 3 Discussion

Primary membranous nephropathy (MN), the predominant cause of idiopathic nephrotic syndrome in non-diabetic adults worldwide, is a significant contributor to renal failure [2,3]. The hallmark clinical feature of MN is nephrotic-range proteinuria accompanied by edema. Approximately two-thirds of patients present with nephrotic syndrome, while the remaining third exhibit asymptomatic proteinuria, typically ≤3.5 g/day. Subtle early symptoms frequently delay diagnosis, resulting in advanced disease and severe renal impairment at presentation, which renders many patients unsuitable as donors.

Kidneys from MN donors pose risks of proteinuria and renal dysfunction, potentially jeopardizing long-term graft survival and function [4]. Pathologically, MN is characterized by diffuse thickening of the glomerular basement membrane thickening due to subepithelial immune complex deposition. While the diagnosis of MN relies on renal biopsy and biomarker detection (e.g., PLA2R antibodies), these assessments are not routinely integrated into donor evaluations, leading to the under-recognition of MN donors. The resulting scarcity of MN donor kidney transplants reported in the literature may therefore be attributed to these diagnostic limitations and clinical risks. Indeed, only seven previous cases of MN donor kidney transplantation are documented (Table. 1). The pathological classifications of all deceased donor kidneys were unknown prior to procurement, with only two living donor kidneys confirmed for MN preoperatively. All donors, except one (serum creatinine: 454 μmol/L), exhibited serum creatinine levels <85 μmol/L and only one donor demonstrated proteinuria (2+). One recipient died from hepatic failure and acute pancreatitis at 20 months post-transplantation. Surviving recipients displayed trace or undetectable proteinuria, with serum creatinine levels ranging from 106.1 to 186.5 μmol/L.

**Table 1:** Previous cases of kidney transplantation from donors with preexisting membranous nephropathy.

Case	1	2	3	4	5	6		7
Author	Parker et al. [5]	Nakazawa et al. [6]	Akioka et al. [7,8]	Mirza et al. [9]	Tsujimura et al. [10]	Molina Andújar et al. [11]		Nuccitelli et al. [12]

Donor								
Age (years)	37	NA	63	36	49	63		41
Sex	Female	NA	Male	Female	Female	Male		Female
Medical history	Hypertension, Gestational Diabetes	NA	MN	Hypertension	MN	Chronic obstructive pulmonary disease		Cosmetic surgery
Main death cause	Extensive cerebral infarction	NA	Living donor	Subarachnoid hemorrhage	Living donor	Respiratory failure		Living donor
Renal biopsy	MN	MN	NA	MN	NA	NA		NA
Proteinuria	No proteinuria	2+	No proteinuria	No proteinuria	No proteinuria	NA		No proteinuria
SCr (μmol/L)	70.7	NA	84.9	N/A	57.5	79.6		79.6
Donor type	Deceased	Deceased	Living	Simultaneous kidney and cardiac transplantation	Living	Deceased		Living

Recipient								
Age (years)	50	41	38	61	50	58	73	60
Sex	Male	Male	Male	Male	Male	Male	Male	Male
Medical history	Type II diabetic nephropathy	NA	NA	Glomerulonephritis	IgA Nephropathy	End-stage kidney disease of unknown origin	End-stage kidney disease of unknown origin	IgA nephropathy

Pre-transplantation								
Proteinuria	NA	NA	NA	1+	NA	NA	NA	NA
SCr (μmol/L)	663.0	NA	NA	265.2	287.3	NA	NA	NA

Post-transplantation								
Renal biopsy	NA	MN	MN	MN	MN	MN	MN	MN
Follow-up Duration (months)	34	20	57	27	18	12	12	13

Renal function at last follow-up								
Proteinuria	NA	NA	No proteinuria	Trace	No proteinuria	NA	NA	Trace
SCr (μmol/L)	106.1	N/A	150.3	124.0	N/A	186.5	150.3	141.4
Outcome	Stable	Died of hepatic failure and acute pancreatitis	Stable	Stable	Stable	Stable	Stable	Stable

MN, membranous nephropathy; SCr, serum creatinine; NA, not available.

We report on two recipients from the same deceased donor, both showing no evidence of graft failure after an 8-year follow-up period. The recipient of the left kidney developed proteinuria exceeding 3 g/day (Fig. 3), which was confirmed via biopsy as acute T cell-mediated rejection (Type IIA) and donor-derived MN. Immunosuppression was intensified with short-term ATG and IVIG, achieving gradual renal recovery. This case is similar to a case reported by Nakazawa et al. [9], where the recipient developed acute rejection after transplantation from an MN donor, with renal function stabilizing after intravenous pulse steroid therapy. Subepithelial deposits induce basement membrane thickening and podocyte foot process effacement, which disrupts the glomerular filtration barrier and may lead to the exposure of cryptic endothelial epitopes or the generation of novel antigens. This process may increase the risk of early post-transplantation acute rejection through the development of endarteritis. In contrast, the recipient of the right kidney experienced an uncomplicated recovery. Protocol biopsies revealed persistent partial podocyte foot process effacement, intramembranous electron-dense deposits, and diminished PLA2R staining intensity, indicating partial pathological regression. Notably, both recipients maintained stable renal function and were free of proteinuria at the 8-year follow-up. Proteinuria is commonly observed in kidney transplant recipients within the first month post-transplantation. Low-level proteinuria is generally considered harmless, and its short-term reduction has been associated with improved long-term graft survival [13].

A potential limitation of this report is the absence of follow-up biopsies after 8 months. While clinically justified by the stable graft function and absence of overt indications for biopsy in both recipients, further histological follow-up would have provided definitive insight into the long-term pathological resolution of donor-derived MN or the persistence of subtle subclinical changes. This lack of serial pathological data limits our ability to fully characterize the long-term progression or regression of donor-derived MN at a microscopic level, despite excellent clinical outcomes. Furthermore, utilizing kidneys from MN donors introduces practical challenges in screening. Precise diagnosis of MN in deceased donor kidneys, requiring detailed histological and immunohistochemical analyses (e.g., electron microscopy, PLA2R staining), is often constrained by time-sensitive organ procurement protocols. These logistical hurdles may limit comprehensive pre-transplant assessment, underscoring the need for rapid, reliable diagnostic methods and careful risk-benefit evaluation during donor selection.

## 4 Conclusion

Kidney transplantations from MN donors may exhibit transient early proteinuria; however, long-term follow-up indicates stable renal function, resolution of proteinuria, and partial pathological remission. Our findings suggest that cautious use of MN donor kidneys may be a viable strategy to expand the donor pool, provided rigorous clinical oversight is implemented. Strategic immunosuppression, meticulous donor-recipient matching, and protocol biopsies are critical for optimizing success. Furthermore, as a case report, our findings cannot be generalized, and large-scale longitudinal studies with standardized histological evaluations remain imperative to definitively establish selection criteria and refine post-transplant management protocols for MN donor utilization.

## Author contribution

Shan-Qun Shen: Conceptualization, Methodology, Software, Data curation, Writing-Original draft.

Wen-Han Peng: Writing-Reviewing and Editing.

## Patient consent

Written informed consent was obtained from the patient for publication of this case report and accompanying images. A copy of the written consent is available for review by the Editor-in-Chief of this journal on request.

## Ethics approval

This study was performed in line with the principles of the Declaration of Helsinki. This study was approved by our institution's Research Ethics Committee.

## Research registration number

Not applicable.

## Funding

This research did not receive any specific grant from funding agencies in the public, commercial, or not-for-profit sectors.

## Declaration of competing interest

The authors declare no conflict of interest.
